# Adsorptive removal of tetracycline by sustainable ceramsite substrate from bentonite/red mud/pine sawdust

**DOI:** 10.1038/s41598-020-59850-2

**Published:** 2020-02-19

**Authors:** Yanting Wang, Shengying Gong, Yazhuo Li, Zhang Li, Jie Fu

**Affiliations:** 1 Department of Environmental Science & Engineering, Fudan University, Shanghai, 200433 China; 2 School of Environmental Science and Engineering, Huazhong University of Science and Technology, Wuhan, 430074 China

**Keywords:** Wetlands ecology, Pollution remediation

## Abstract

In this study, a novel, sustainable and efficient ceramsite substrate of constructed wetlands (CWs) were prepared for tetracycline (TC) removal by employing bentonite (Ben) and red mud (Rm) as the main materials and pine sawdust (Ps) as the additive. The optimal parameters for Ben/Rm/Ps ceramsite preparation were obtained via orthogonal and one-factor experimental designs, and the optimal parameters were presented as follows: mass ratio of Ben: Rm: Ps = 4:1:0.9, preheating temperature = 240 °C, preheating time = 20 min, calcining temperature = 1150 °C, and calcining time = 14 min. The properties of Ben/Rm/Ps-op ceramsite (obtained at the optimal condition) were first analyzed, including XRD and SEM, and demonstrated a microporous structure with some crystal strength components. Neutral condition and higher temperature were indicated conducive to improve the TC removal efficiency, while coexisting ions (Na^+^ or Ca^2+^) showed adverse effect for TC adsorption by Ben/Rm/Ps-op. In addition, adsorption kinetics and isotherm could be well described by the second-order kinetics and linear isothermal model, respectively, which suggested chemisorption and multilayer adsorption thickness increased infinitely. The theoretical maximum TC adsorption capacity of Ben/Rm/Ps-op at 20 °C reached up to 2.5602 mg/g. In addition, Ben/Rm/Ps-op could effectively remove TC as the CWs substrate under a dynamic flow condition. Further, Ben/Rm/Ps-op exhibited high reusability capability and stability for TC removal, and the adsorption amount still remained for 2.13 mg/g (*C*_0_ = 80 mg/L) after three consecutive cycles.

## Introduction

As the most widely used spectrum antibiotics, tetracycline (TC) can effectively inhibit the activities of chlamydia, mycoplasma, protozoan parasites, rickettsiae, gram-negative bacteria and gram-positive bacteria^1^. Therefore, it is widely and frequently applied in medical and health, poultry breeding and other industries for disease treatment^2^. Nevertheless, animals usually fail to fully metabolize TC, and most of ingested TC are discharged into the environment, which would pose potent pollution danger to environment consequently^3–6^. Long-term exposure to TC in the environment has caused serious concern about toxicity and bacterial resistance, which leads to ecological damage and threatens human health through bioaccumulation in the food chain^7^. It has been proved that TC can interact with phospholipids and be adsorbed by biological cell membranes, which can also lead to complications of human allergic reactions. Despite of short half-life of TC, the current sewage treatment system can not completely remove TC^4^, which has caused reports of TC residues in surface water, groundwater and soil around the world, and even in many foods^7^. Therefore, it is of great significance to develop effective methods to remove TC in water environment.

To date, there are a lot of techniques such as filtration, ion exchange, adsorption, electrochemical treatment, membrane separation, catalytic degradation, etc. that have been developed for the removal of antibiotics, toxic heavy metals and dye pollutants from wastewater^8–10^. Among them, adsorption is one of the most reliable methods due to its ease of operation, low process cost, high removal efficiency and regeneration ability^11^. Many prepared adsorbents have been reported for the removal of pollutants in water, for example, the removal of As (V)^12^ and Cr (VI)^13–15^ by prepared adsorbent with high removal rates. Therefore, this study intends to employ adsorption mechanism to remove TC.

With the emergence of constructed wetlands (CWs) called “earth artificial kidney”, people are more inclined to use this ecological method to further treat tail water of municipal wastewater treatment plant. In addition, CWs were designed and built to take advantage of natural processes involving wetland vegetation, soil and associated microbial combinations^16^, which was recognized as a relatively natural, low-cost, energy-efficient, and ecological technology for treating municipal or domestic wastewaters^17^. CWs are mainly purified by the synergy of microorganisms, plants and substrates. They often showed better removal efficiency for nitrogen, phosphorus and organic compounds, etc. Wetland substrate is the skeleton of wetland ecosystem, which is generally composed of gravel, sand, soil and artificial matrix, accounting for almost 80% of the total volume of wetland. The efficiency and effect of constructed wetland water treatment will be greatly improved by using the filler matrix with good selectivity.

Preparing ceramic products via high temperature calcination of raw materials is a promising method to improve the performance and safety of materials and popularize applications. Ceramic production can bring a lot of benefits to the product, such as improving mechanical strength by curing/stabilization; stabilizing harmful metals by adding embedded crystals; removing organic pollutants/pathogenic agents by pyrolysis and gasification; and improving adsorption capacity by generating microporous structures^18–20^. Bentonite has a good adsorption capacity for pollutants, and can be used to remove petroleum toxins, purify gasoline and kerosene, and treat wastewater. The main active mineral ingredient of bentonite is montmorillonite with high grade content of 85–90%. High content of SiO_2_ and Al_2_O_3_ makes bentonite meet the requirement for sintering ceramsite as the main material^21^. However, low fusing composition (8.33%) can not guarantee the good performance of ceramics with bentonite as the only material, while more energy will be consumed and costs increase. Therefore, it is necessary to mix other materials to improve the performance of sintered ceramics, reduce energy consumption and reduce production costs.

Red mud, a polluting waste residue discharged from alumina extraction in aluminium industry, contains a large amount of iron oxide. In addition, its main minerals are aragonite and calcite, with the content of 60–65%. Mixing red mud into bentonite for ceramsite preparing could bring two benefits: (1) high content of Al_2_O_3_ contributes to forming more mullite with high strength and further enhancing the mechanical strength of ceramsite; (2) more melting aids can improve the firing properties of ceramsite.

Further, in order to remove TC efficiently, ceramsite requires strong adsorption capacity, which is closely related to its specific surface area and internal porosity. Pine sawdust (Ps), as we all know, can produce CO, CO_2_ and other gases by pyrolysis at high temperature, which would improve the expansion and porosity of ceramsite. Therefore, Ps may act as a functional additive to improve the sintering process and properties of Ben/Rm ceramsite.

At present, some studies have reported the use of red mud with other raw materials such as fly ash, clay and other fired ceramics or ceramic bricks^22,23^. Especially, Zhang *et al*. demonstrated ceramsite prepared from red mud, bagasse, powdered glass and molasses alcohol wastewater an effective and regenerable material used for TC adsorption treatment^24^. However, bentonite/red mud/pine sawdust (Ben/Rm/Ps) ceramsite has not been reported yet.

To address this knowledge gap, this study prepared Ben/Rm ceramsite and investigated the manufacture parameters through orthogonal experimental designs. Ps, as a functional additive, was then added to the recipe and the optimal parameters were ascertained via one-factor experimental design. In addition, the TC adsorption capacity of Ben/Rm/Ps ceramsite was explored, as well as adsorption mechanism. As such, the overall goal of this study was to develop a stable ceramsite with a good adsorption capacity of TC, which can improve TC immobilization of CWs substrate and extend its lifetime.

## Materials and Methods

### Materials

All chemicals applied in this study were of analytical grade or higher. Sodium hydroxide (NaOH) and hydrochloric acid (HCl) were obtained from Shanghai Bio-Chem Technology Co., Ltd (Shanghai, China). TC solution was prepared from TC hydrochloride power, and TC hydrochloride with a molecular formula of C_22_H_25_ClN_2_O_8_, and molecular weight of 480.90 g/mol was purchased from Aladdin Bio-Chem Reagent Company (Shanghai, China). Bentonite (Ben) was purchased from Jushi mining Company (Baoding, Hebei, China). Red mud (Rm) was collected from waste of Weiqiao Pioneer Group Co., Ltd. (Binzhou, Shandong, China). Pine sawdust (Ps) was obtained from pine branches (Shanghai, China). Deionized (DI) water (18.25 MΩ∙cm) was come into being from a water purification system (EMD Millipore Corp., Merck KGaA, Darmstadt, Germany).

### Preparation of ceramsite

Three raw materials (Ben, Rm and Ps) were dried at 110 °C for 3 h and passed through an 80 mesh sieve. Raw materials mixture with different mass ratio was mixed with running water (30–40 wt%), stirred evenly and twisted into 6–8 mm pellets, then dried at 105 °C for 2 h. The ceramic blank pellets subsequently undergone preheating (120–240 °C) for 15–30 min and roasting (1000–1150 °C) for 5–30 min successively with a KSL-1200 × -S box type high temperature sintering furnace (Hefei Kejing Materials Technology Co., Ltd., Anhui, China). After cooling, the finished ceramsite was obtained.

### Materials characterization

Elemental analysis of Ben and Rm was determined by X-ray fluorescence spectrometry (XRF) using a Spectro Midex system (Spectro Analytical Instrument Company, Germany). Elemental analysis of Ps was detected through a Vario EL III system (Elementar, Germany). The crystal phases of the materials were analyzed using an X’Pert PRO MRD/XL system (XRD) (Panalytical, Almelo, the Netherlands). The surface morphologies of materials were imaged using a scanning electron microscope (SEM) (JSM-840A electron microscope, JEOL, Tokyo, Japan). Thermogravimetric analysis (TG) was conducted on a TA instrument (Netzsch, Selb, Germany). The bulk density and apparent density of ceramsite were determined by GB/T 17431.1-2010 standard method^25^. The compressive strength of ceramsite was measured according to the reported method^26^. The toxicity analysis of heavy metal leaching was carried out using HJ/T 299–2007 method^27^, and GB 3838-2002 standard^28^ and GB 5085.3-2007 standard^29^ were employed as the evaluation criteria.

### Static adsorption experiments

All the adsorption experiments were carried out in dark using brown glass vials (total volume = 150 mL) with 50 mL TC solution and ceramsite on an HZQ-120H heating oscillator (Yiheng Scientific Instrument Co., Ltd., Shanghai, China) with a speed of 160 rpm. pH was adjusted using dilute HCl and NaOH aqueous solution (aq.).

TC adsorption kinetics studies were conducted at pH = 7 and temperature = 20 °C with an initial concentration of TC of 80 mg/L and ceramsite dosage of 20 g/L. At predetermined times (5–600 min), the vials were sacrificially sampled. Besides, to investigate adsorption thermodynamics, the adsorption kinetics assays were carried out at 30 °C and 40 °C as well. For TC adsorption isotherm experiment, the initial concentration of TC was varied from 2 to 80 mg/L with a fixed ceramsite dosage of 20 g/L, and the mixture (pH = 7) was shaken for 24 h at 20 °C to reach the adsorption equilibrium. To explore effect of ceramsite dosage on adsorption, different doses of ceramsite (5–50 mg/L) were added into TC solution (80 mg/L), and the mixture (pH = 7) was shaken for 24 h at 20 °C. To probe effect of pH on adsorption, the equilibrium tests were conducted with an initial TC concentration of 80 mg/L, a ceramsite dosage of 20 g/L, and finial solution pH 2–10 at 20 °C for 24 h. To examine effect of ionic strength, 0–0.25 mol/L NaCl or CaCl_2_ were added into TC solution (80 mg/L) with ceramsite dosage of 20 g/L at pH = 7, temperature of 20 °C and shaking for 24 h. After adsorption is completed, the solution was filtered through a 0.22 μm microfiltration membrane. The concentrations of TC were detected via SP-756P Ultraviolet-Visible Spectrophotometer at 355 nm.

The adsorption amount at predetermined time *t* (*q*_*t*_, mg/g) and equilibrium adsorption amount (*q*_*e*_, mg/g) of TC on materials and removal efficiency (R, %) were calculated via:

1 $${q}_{t}=\frac{({C}_{0}-{C}_{t})V}{m}$$

2 $${q}_{e}=\frac{({C}_{0}-{C}_{e})V}{m}$$

3 $$R=\frac{({C}_{0}-{C}_{{\rm{e}}})}{{C}_{0}}\times 100 \% $$

where *C*_*t*_ (mg/L) is the residual concentration in the liquid phase at sampling time *t* (min); *C*_0_ and *C*_*e*_ (mg/L) are the initial and equilibrium concentrations of TC, respectively; *V* (L) is the total volume of the solution; and *m* (g) is the mass of ceramsite.

TC concentration in the solution phase (*C*_*d*_, mg/L) was determined upon centrifugation and filtration, and the percent of TC desorbed as calculated via:

4 $$D=\frac{{C}_{d}}{({C}_{0}-{C}_{{\rm{e}}})}\times 100 \% $$

### Dynamic column experiments

To better appraise the adsorption efficiency of Ben/Rm/Ps-op for TC, dynamic column experiments were carried out. A glass column wrapped in aluminium foil was employed in the tests, with a height of 50 cm and an internal diameter of 5 cm. Ceramsites were loaded into the column with a height of 20 cm, and the corresponding filter volume was 393 mL. The initial concentration of TC was set as 4 mg/L, and the solution was bumped into the column in an up-flow mode. For the effect of HRT, column tests were carried out at different HRTs (5, 10 and 15 h), and the solution samples were collected daily for detecting TC concentration. For the effect of packing height, the column experiments were conducted with different packing heights (10, 15 and 20 cm) at HRT of 0.5 h, and the solution samples were collected daily for detecting TC concentration.

### Ceramsite regeneration studies

Regeneration and recyclability are crucial parameters for industrial application of adsorbents. In this study, regeneration of ceramsite was investigated in a batch test. The used adsorbent Ben/Rm/Ps-op were regenerated by washing with N, N-dimethylformamide (DMF) several times and freeze-drying for 24 h. The adsorption-regeneration experiment was repeated 3 times in 80 mg/L TC solution at 20 °C and pH = 7.

## Results and Discussion

### Preparation and optimization of Ben/Rm ceramsite

**Figure 1:**
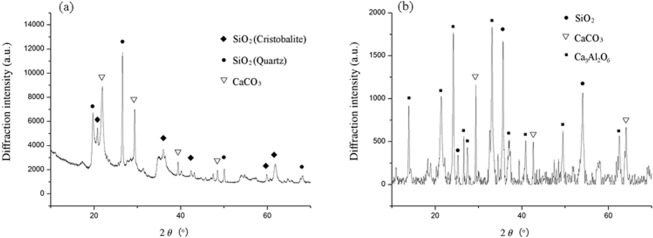
XRD spectra of bentonite (**a**) and red mud (**b**).

Elemental compositions of Ben and Rm were determined by XRF (Table 1). High contents of SiO_2_ and Al_2_O_3_ were found in Ben (SiO_2_ = 60.8% and Al_2_O_3_ = 11.9%) and Rm (SiO_2_ = 15.2% and Al_2_O_3_ = 22.2%), which are sufficient to meet the requirements of sintering ceramsite^21^. In addition to SiO_2_ and Al_2_O_3_, some fluxing components (e.g., MgO, CaO, Na_2_O, and K_2_O) and volatiles also present in Ben and Rm. About 10.48% and 50.68% of fluxing components are respectively contained in Ben and Rm. Therefore, Ben and Rm can be used as the main materials for firing ceramsite. Moreover, the mineral constituent of Ben and Rm were appraised by XRD. For Ben, the peak strength of SiO_2_ crystal phase is most intense, and crystal peaks of CaO_3_ can also be observed (Fig. 1a). Since other major components of Ben such as Al_2_O_3_ and MgO did not form crystal morphology, corresponding peaks were not detected. For Rm, large amount of crystal SiO_2_ and a certain amount of CaCO_3_ and Ca_3_Al_2_O_6_ were detected (Fig. 1b).

**Table 1:** Chemical composition (wt.%) of Ben and Rm, as obtained by XRF.

	Ben	Rm		Bentonite	Rm
SiO_2_	60.8000	15.2000	ZnO	0.0070	0.0090
Al_2_O_3_	11.9000	22.1600	CuO	0.0039	0.0120
MgO	4.0000	0.2000	Nb_2_O_5_	0.0026	0.0125
CaO	3.9200	3.3800	Rb_2_O	0.0025	/
Fe_2_O_3_	1.1400	34.7100	Y_2_O_3_	0.0016	0.0107
Na_2_O	0.9750	12.3000	P_2_O_5_	/	0.3830
K_2_O	0.4450	0.0903	Cr_2_O_3_	/	0.1190
TiO_2_	0.1750	6.1960	CeO_2_	/	0.0440
SrO	0.0535	0.0201	Gd_2_O_3_	/	0.0270
MnO	0.0378	0.0857	NiO	/	0.0090
SO_3_	0.0315	0.5770	As_2_O_3_	/	0.0090
ZrO_2_	0.0173	0.1810	Ga_2_O_3_	/	0.0080
Cl	0.0150	0.0630

According to elemental compositions in Table 1, we calculated the content of SiO_2_, Al_2_O_3_, fluxing components and SiO_2_ + Al_2_O_3_ in the mixture of Ben and Rm with different ratios (1:1, 2:1, 3:1, 4:1, 5:1, 6:1 and 7:1) (Table 2). With the increase of proportion (Ben: Rm), the content of SiO_2_ and SiO_2_ + Al_2_O_3_ would increase, while the content of Al_2_O_3_ and fluxing components would decrease. Taking into account Riley three-phase diagram^30^ and the actual situation of adding as much Rm as possible to the raw materials, four ratios (3:1, 4:1, 5:1 and 6:1) were adopted to prepare Ben/Rm ceramsite.

**Table 2:** The content of SiO_2_, Al_2_O_3_, fluxing components and SiO_2_ + Al_2_O_3_ in the mixture of Ben and Rm with different ratios (1:1, 2:1, 3:1, 4:1, 5:1, 6:1 and 7:1).

Mass ratio of Ben: Rm	SiO_2_ (%)	Al_2_O_3_ (%)	Fluxing components (%)	SiO_2_ + Al_2_O_3_ (%)
1:1	38	17.03	30.58	55.03
2:1	45.6	15.32	23.88	60.92
3:1	49.4	14.465	20.53	63.865
4:1	51.68	13.952	18.52	65.632
5:1	53.2	13.61	17.18	66.81
6:1	54.286	13.366	16.22	67.652
7:1	55.1	13.1825	15.505	68.2825

To optimize the preparation of CFA/WS ceramsite and identify the critical factors of determining the ceramsite properties, an orthogonal experimental design of five factors (mass ratio of Ben: Rm, preheating temperature and time, calcining temperature and time) and four levels (L_16_(4)^5^) were conducted and bulk density of ceramsite was employed as the evaluation index. Relatively lower bulk density is preferable, which demonstrates higher porous ceramsite bodies^31^. The significance levels of different influencing factors on the ceramsite bulk density were clarified through the range analysis^32^. Table 3 summed up the results of L_16_(4)^5^ orthogonal design. The *K* value for each level of a parameter was the average of four bulk density values, and the range value (*R*) for each factor was the difference between the maximal and minimal *K* values of the four levels. The range analysis suggested Ben: Rm ratio was the most important factor and followed by preheating time and calcining temperature, while others factors were not of significance.

**Table 3:** L_16_(4)^5^ orthogonal experimental design for sintering Ben/Rm ceramsite.

Exp. No.	Ben:Rm	Preheating temp. (°C)	Preheating time (min)	Calcining tem. (°C)	Calcining time (min)	Bulk density (kg/m^3^)
1	3:1	120	15	1000	5	778.19
2	3:1	160	20	1050	8	773.25
3	3:1	200	25	1100	11	742.53
4	3:1	240	30	1150	14	672.12
5	4:1	120	20	1100	14	645.26
6	4:1	160	15	1150	11	653.49
7	4:1	200	30	1000	8	689.82
8	4:1	240	25	1050	5	659.10
9	5:1	120	25	1150	8	658.69
10	5:1	160	30	1100	5	674.52
11	5:1	200	15	1050	14	678.33
12	5:1	240	20	1000	11	682.27
13	6:1	120	30	1050	11	574.97
14	6:1	160	25	1000	14	621.93
15	6:1	200	20	1150	5	541.29
16	6:1	240	15	1100	8	595.20
k_1_	741.52	664.28	593.80	695.30	663.28	
k_2_	661.92	680.80	660.52	671.41	679.24	
k_3_	673.45	662.99	670.56	664.38	663.32	
k_4_	583.35	652.17	652.86	661.34	654.41	
R	158.17	28.63	76.76	33.96	24.83	

As depicted in Table 3, with the increase of Ben: Rm ratio, the bulk density of ceramic displayed a decline trend, which may be related to the change of sintering and volatile compositions. In addition, when the preheating time was within 15–25 min, ceramsite bulk density increased obviously with the increase of time. However, increasing the preheating time from 25 to 30 min, the bulk density showed a slightly decrease. Under certain conditions, increasing preheating time facilitated softening ceramsite, which ensured ceramsite produced enough gas in the roasting stage, thus reduced the bulk density. However, when preheating time is too long, organic matter and carbonate will decompose and volatilize to produce gas in the preheating stage, which will reduce the gas amount produced in roasting stage, then the bulk density increased.

### Preparation and optimization of Ben/Rm/Ps ceramsite

**Figure 2:**
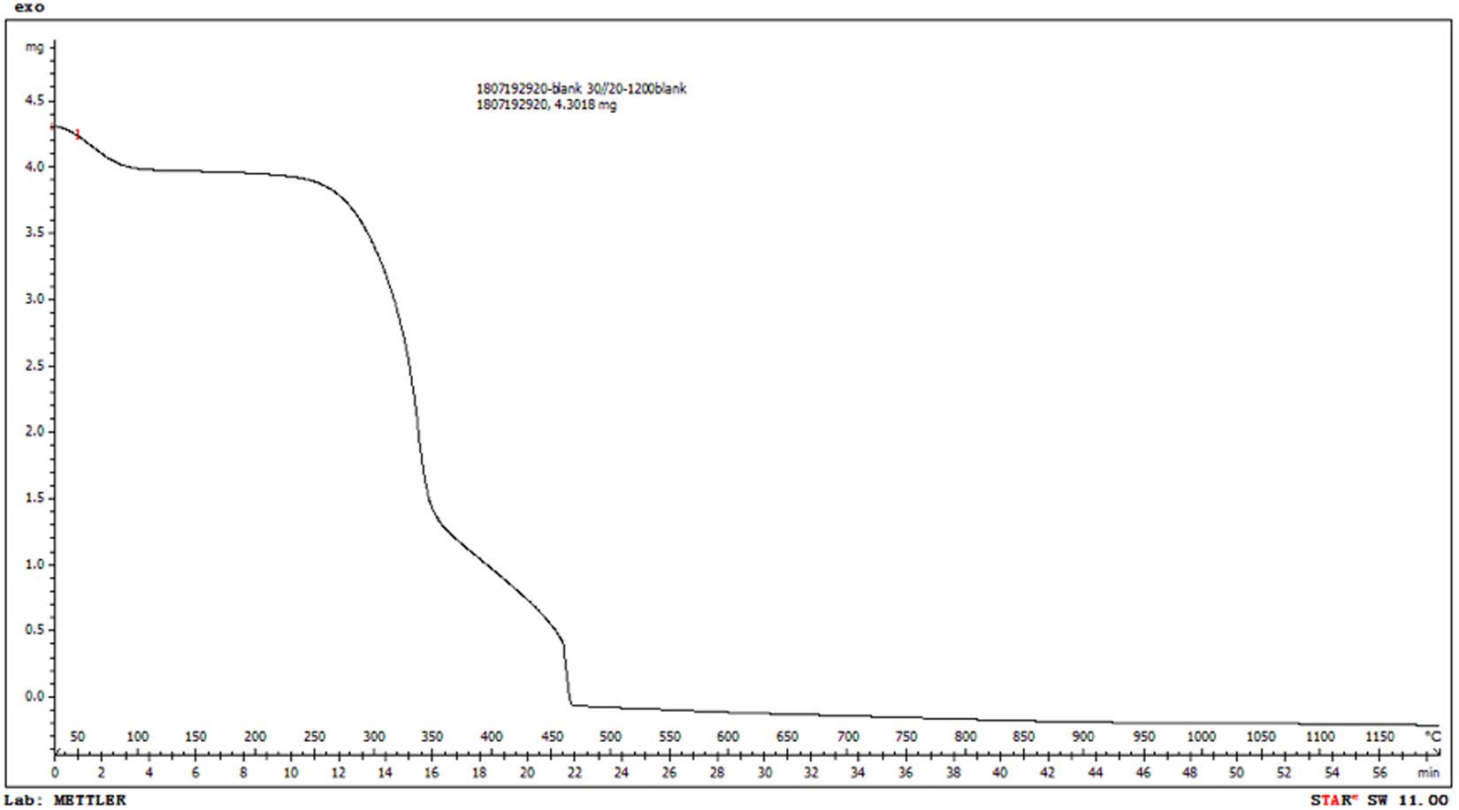
Thermogravimetric (TG) analysis of pine sawdust.

Ps was characterized by ultimate analysis (Table 4) and TG (Fig. 2). The TG curve shows that the Ps mass nearly did not change below 260 °C, while a drastic decline occurred within 260–468 °C, and above 468 °C little Ps was residual (Fig. 2). Therefore, decomposition of Ps is expected to occur in the initial stage of ceramic calcination stage, which can improve the sintering performance and promote the formation of ceramic porous structure.

**Table 4:** Ultimate analysis data of pine sawdust (wt%).

C	H	O	N	S
46.48	6.055	43.45	0.044	0.121

The aforementioned L_16_(4)^5^ orthogonal experimental design indicated the mass ratio of raw materials and preheating time were the most significant influence factors on ceramic sintering. Therefore, for the preparation of Ben/Rm/Ps ceramsite, the mass ratio of raw materials and preheating time were optimized by using one-factor experimental design, while the other fixed parameters were adopted the optimal ones based on L_16_(4)^5^ results, i.e., preheating temperature = 240 °C, calcining temperature = 1150 °C, calcining time = 14 min.

**Figure 3:**
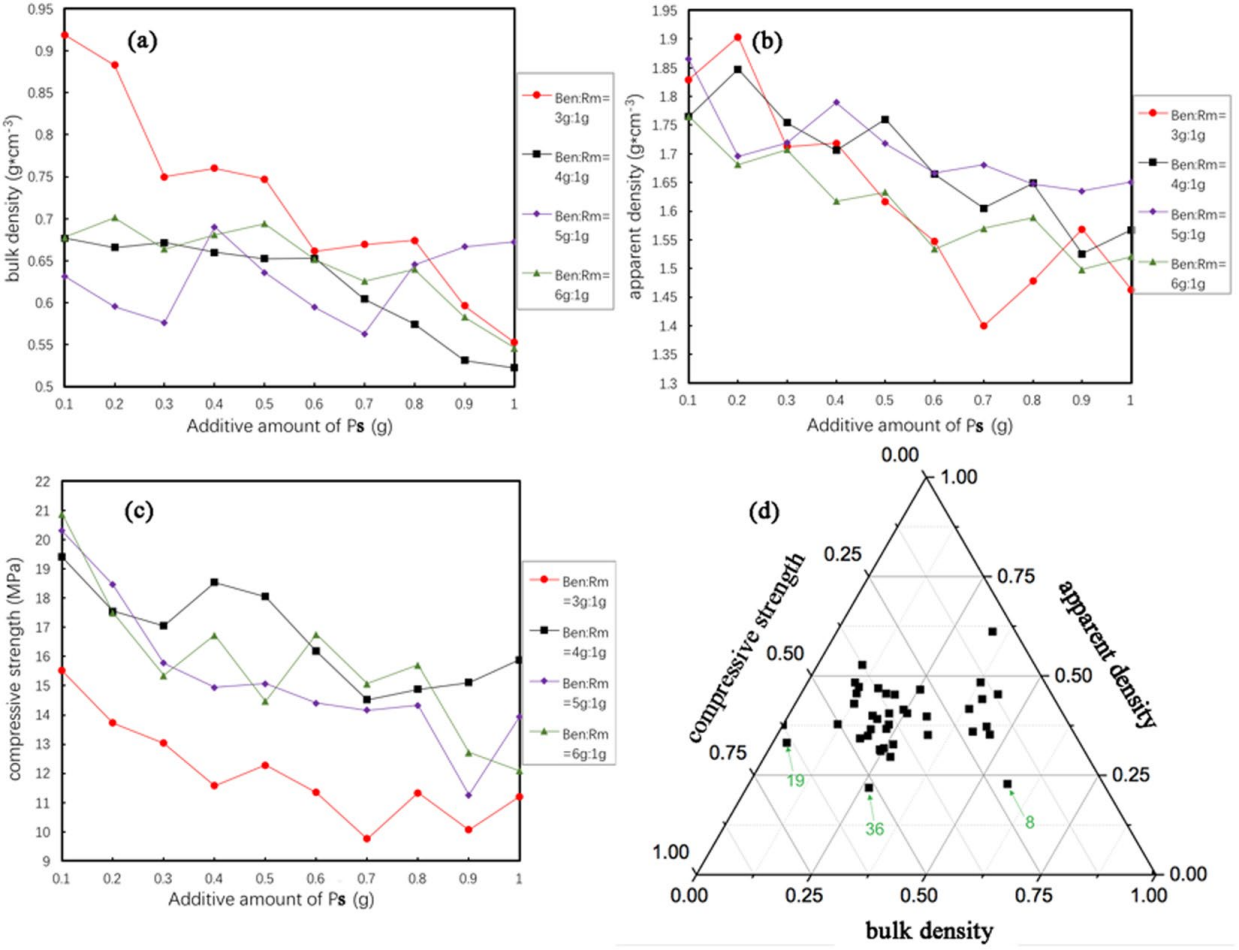
Optimizaiton of mass ratio of raw materials for preparation of Ben/Rm/Ps ceramsite. Bulk density (**a**), apparent density (**b**), compressive strength (**c**) and three-phase diagram based on the three parameters (**d**) of Ben/Rm/Ps ceramsite obtained at different raw materials ratio.

For the optimization of mass ratio of raw materials, the preheating time was fixed at 15 min, and four levels of Ben: Rm were employed, i.e., 3 g:1 g, 4 g:1 g, 5 g:1 g and 6 g:1 g. Different amounts of Ps (0.1–1 g) were added to the raw materials to sinter ceramsite. Apparent density, bulk density and compressive strength of ceramsite were determined to appraise the influence of raw materials ratio (Fig. 3a–c). Besides, a three-phase diagram based on the three parameters was presented in Fig. 3d, during which 19, 36 and 8 represent mass ratios of Ben: Rm: Ps = 4:1:0.9, 6:1:0.6 and 3:1:0.8, respectively. Combining the three-phase diagram (Fig. 3d) with apparent density, bulk density and compressive strength curves (Fig. 3a–c), 8, 19 and 36 displayed outstanding performance among all ratios. Besides, 19 and 36 have little difference in compressive strength and apparent density, while showed much more excellent than 8. In addition, comparing with 36, 19 exhibited smaller bulk density and possessed more red mud, which is conducive to waste utilization. Therefore, the Ben: Rm: Ps ratio of 4:1:0.9 was chosen for the follow-up experiment.

**Figure 4:**
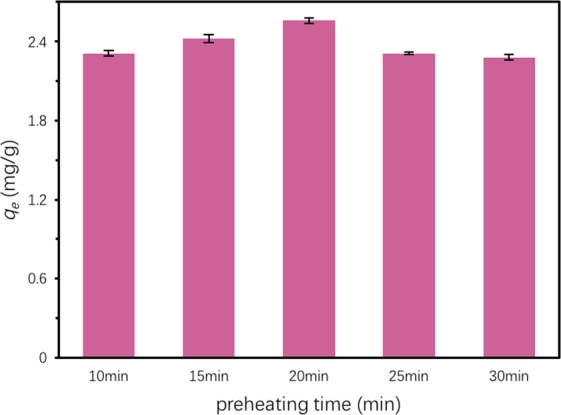
TC adsorption capacity of Ben/Rm/Ps ceramsite obtained at different preheating time. Adsorption condition: total TC = 80 mg/L, adsorbents dose = 20 g/L, *T* = 20 °C, pH = 7 and equilibrium time = 24 h.

For the optimization of preheating time, the optimal ratio of Ben: Rm: Ps (4:1:0.9) was employed and the preheating time was varied from 10 min to 30 min. TC adsorption capacity of obtained ceramsite was assessed by static adsorption experiments. The TC adsorption capacity of ceramsite showed a trend of firstly increasing and then decreasing with the increase of preheating time (Fig. 4). From 10 min to 20 min, the TC adsorption capacity of ceramsite showed a steady increase, and reached the maximum value at 20 min. With the increase of preheating time, softening degree of ceramsite raises, which is conducive to ensuring enough gas producing in the roasting stage, thus reducing bulk density. Continuously increase the preheating time from 20 min to 30 min, the TC adsorption capacity of ceramsite displayed a quick decline. This can be explained that the organic matter and carbonate will decompose and volatilize to produce gas in the preheating stage for too long preheating time, which will reduce the amount of gas produced in the roasting stage and increase the stacking density.

To sum up, the optimal parameters for preparation of Ben/Rm/Ps ceramsite were determined as: Ben: Rm: Ps = 4:1:0.9, preheating temperature = 240 °C, preheating time = 20 min, calcining temperature = 1150 °C, and calcining time = 14 min. The Ben/Rm/Ps ceramsite prepared at this optimal condition (hereafter referred to as Ben/Rm/Ps-op) had an apparent density of 1.41 g/cm^3^, bulk density of 0.54 g/cm^3^ and compressive strength of 19.45 MPa.

**Figure 5:**
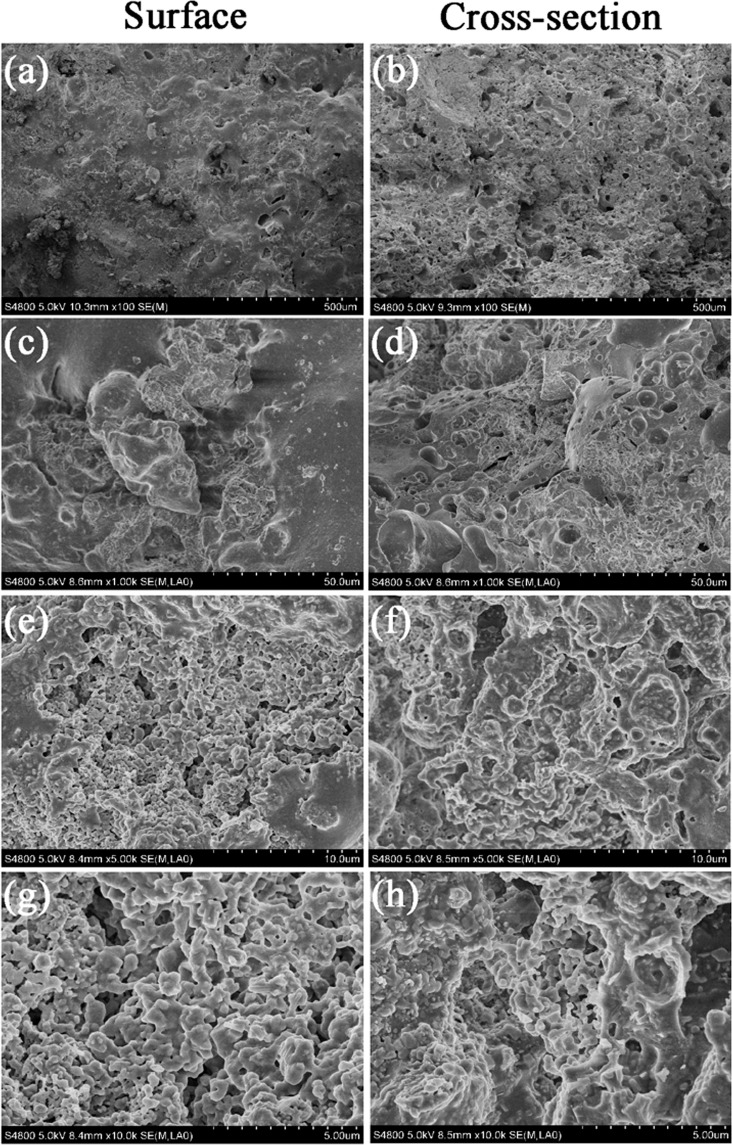
SEM images of Ben/Rm/Ps-op ceramsite surface at ×100 (**a**), ×1000 (**c**), ×5000 (**e**) and ×10000 (**g**); SEM images of Ben/Rm/Ps-op ceramsite cross-section at ×100 (**b**), ×1000 (**d**), ×5000 (**f**) and ×10000 (**h**).

**Figure 6:**
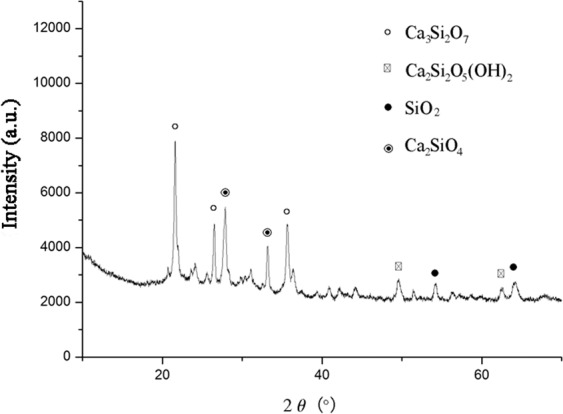
XRD profiles of Ben/Rm/Ps-op ceramsite.

SEM analysis (Fig. 5) showed that both the surface and cross-section of Ben/Rm/Ps-op was mainly distributed by macroporous and microporous structures with different sizes, indicating a large specific surface area and widely distributed adsorption sites. The SEM results demonstrated that ceramsite was a porous ceramsite and might be a good adsorbent for wastewater treatment. XRD detected the formation of several crystal phases in Ben/Rm/Ps-op including SiO_2_, Ca_3_Si_2_O_7_, Ca_2_Si_2_O_5_(OH)_2_ and Ca_2_SiO_4_ (Fig. 6). The crystal compositions are helpful to improve the strength of ceramsite; the active components such as SiO_2_, Si_2_O_7_^6−^, Si_2_O_5_(OH)_2_^4−^ and SiO_4_^4−^ can be adsorption sites of TC via ion exchange. Since TC contain a positively charged group in the structure, regardless of the zero net charge or negative net charge, it is likely that the molecule arranges at the surface in such a way that the positively charged group is located very close to the surface. SiO_2_ is negatively charged at the solution of pH > 2.5, which could easily combine with TC. In addition, negatively charged groups Si_2_O_7_^6−^, Si_2_O_5_(OH)_2_^4−^ and SiO_4_^4−^ also showed excellent binding ability with TC.

Moreover, heavy metal leaching toxicity analysis (Table 5) revealed the concentrations of leached heavy metals from Ben/Rm/Ps-op were far below hazardous wastes standard (GB 5085.3-2007)^29^, and basically met the surface water quality of Class III (GB 3838-2002)^28^, recommending Ben/Rm/Ps-op will not cause secondary pollution to the aquatic environment. Therefore, Ben/Rm/Ps-op is a safe ceramsite with great mechanical strength and adsorption capacity.

**Table 5:** Heavy metal leaching toxicity analysis of Ben/Rm/Ps-op ceramsite.

Heavy metals	Cu	Ni	Pb	Cr	Zn	Cd
Detected concentration (mg/L)	0.006	0.002	ND^a^	0.025	0.004	ND
GB 5085.3-2007 standard (mg/L)	100	5	5	15	100	1
GB 3838-2002 standard (mg/L)	1	ND	0.01	0.05	1	0.005

^a^ND = Not detected.

### Adsorption of TC

#### Adsorption kinetics

**Figure 7:**
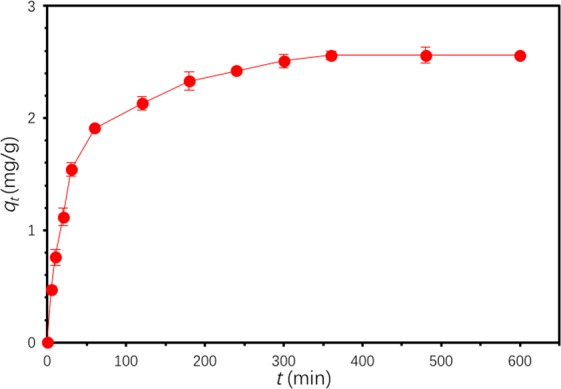
Adsorption kinetic of TC by Ben/Rm/Ps-op ceramsite. (*C*_0_ = 80 mg/L, *V* = 50 mL, speed = 160 rpm, adsorbent dose = 20 g/L, *T* = 20 °C, pH = 7).

It is observed in Fig. 7 that Ben/Rm/Ps-op could catch most of TC from aqueous solution in the initial 180 min and reach equilibrium after 360 min. After calculation, 64% TC was removed at equilibrium and the equilibrium adsorption capacity was as high as 2.5602 mg/g.

Pseudo-first-order and pseudo-second-order models were tested to analyze the kinetics results, which are expressed as^33,34^:

Pseudo-first-order model:

5 $$\mathrm{ln}\,{q}_{e}-{q}_{t}=ln{q}_{e}-{k}_{1}t$$

**Figure 8:**
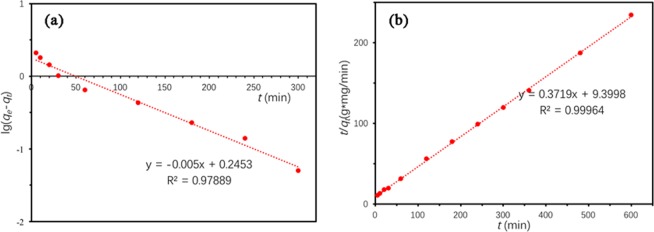
Kinetics plots for TC adsorption on Ben/Rm/Ps-op. (**a**) pseudo-first-order model; (**b**) pseudo-second-order model.

Pseudo-second-order model:

6 $$\frac{t}{{q}_{t}}=\frac{1}{{k}_{2}{{q}_{e}}^{2}}+\frac{t}{{q}_{e}}$$

where *q*_*t*_ and *q*_*e*_ (mg/g) are the adsorption capacities of TC at time *t* (min) and equilibrium, respectively; *k*_1_(min^−1^) is the rate constant for pseudo-first-order model and *k*_2_(g/(mg∙min)) is for pseudo-second-order model, respectively. As a result, the pseudo-second-order model showed higher R^2^ = 0.9996, compared with R^2^ = 0.9789 for the pseudo-first-order model (Table 6, Fig. 8). Besides, *q*_*e,cal*_ (2.6889 mg/g) of pseudo-second-order model was closer to *q*_*e,exp*_ value (2.5602 mg/g) than *q*_*e,cal*_ (1.7591 mg/g) of pseudo-first-order model did. Consequently, the adsorption of TC by Ben/Rm/Ps-op conforms to pseudo-second-order model, indicating that the rate controlling step for adsorption was a chemical interaction^35^.

**Table 6:** Kinetic model parameters for adsorption of TC by Ben/Rm/Ps-op ceramsite.

Model	Parameter	Ben/Rm/Ps-op ceramsite
Pseudo-first-order model	*q*_*e,cal*_^a^ (mg/g)	1.7591
	*k*_1_ (min^−1^)	0.0115
	*R*^2^	0.9789
Pseudo-second-order model	*q*_*e,cal*_^a^ (mg/g)	2.6889
	*k*_2_ (g/(mg∙min))	0.0147
	*R*^2^	0.9996
	*q*_*e,exp*_^b^ (mg/g)	2.5602

^a^Calculated adsorption capacity from kinetic models.

^b^Observed adsorption capacity form experiments.

#### Adsorption mechanism

The pseudo-second-order model, including all processes of adsorption (external liquid film diffusion, surface adsorption, intraparticle diffusion and so on), could not accurately reflect the mechanism of this adsorption process^36^. For further exploring adsorption mechanism of TC on Ben/Rm/Ps-op, intraparticle diffusion model was employed to determine the type of rate-controlling step. This model can be delivered as follows:

7 $${q}_{t}={k}_{int}{t}^{0.5}+Const$$

where *k*_*int*_ (mg/(g∙min^0.5^)) is the constants for the intraparticle diffusion model, and *Const* (mg/g) is a constant proportional to the extent of boundary layer thickness.

**Figure 9:**
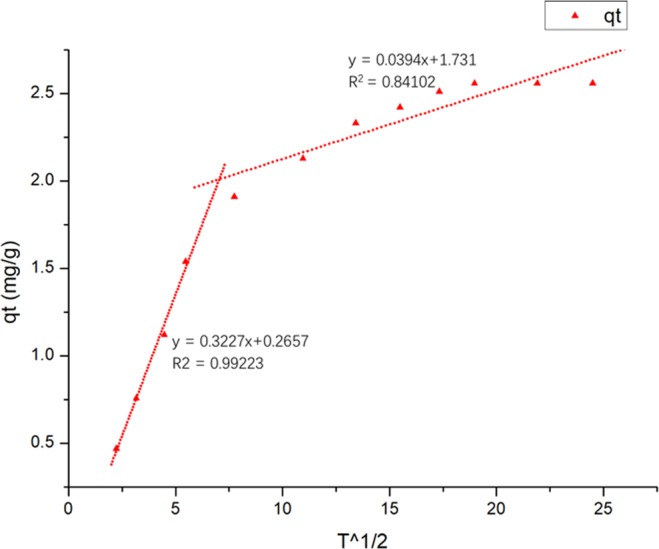
Intraparticle diffusion model for the adsorption of TC on Ben/Rm/Ps-op (*k*_int*1*_ = 0.3227, *k*_int*2*_ = 0.0394).

Figure 9 expressed the linear plots involving two adsorption stages with different slopes. *k*_int*1*_ refers to the external adsorption rate constant in first step, and *k*_int*2*_ indicates the internal adsorption rate constant of the second stage by diffusion between particles into the adsorbent. The value of *k*_int*1*_ was higher than that of *k*_int*2*_ owing to a rapid increase in adsorption during the initial phase, with increased active sites available. This result is related to changes in mass transfer rate during adsorption process. The linear portion did not pass through the origin, suggesting that the adsorption mechanism of the TC onto Ben/Rm/Ps-op is not only restrained by the intraparticle diffusion step^37^.

#### Adsorption isotherm

**Figure 10:**
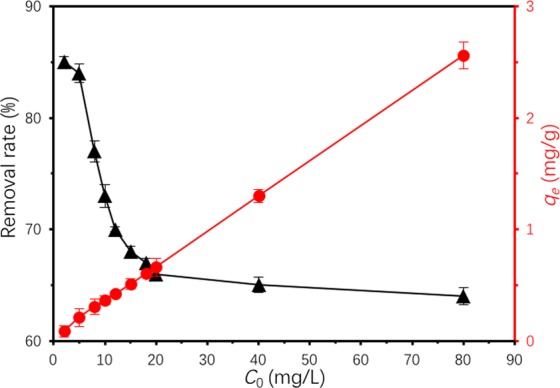
Adsorption isotherms of TC by Ben/Rm/Ps-op (*C*_0_ = 2–80 mg/L, *V* = 50 mL, speed = 160 rpm, adsorbent dose = 20 g/L, *T* = 20 °C, pH = 7, *t* = 12 h).

Adsorption isotherm result at 20 °C was presented in Fig. 10. With the increase of the initial concentration of TC, adsorption capacity of Ben/Rm/Ps-op for TC increased overtly, while TC removal rate significantly descended. The isotherm results were further analyzed using linear, Langmuir, Freundlich, Tempkin and D-R (Dubinin-Radushkevich) isotherm models, as expressed below^38,39^:

The linear isotherm model indicates that amount of adsorption is linearly proportional to the equilibrium solution concentration, which can be depicted as:

8 $${q}_{e}={K}_{d}{C}_{e}$$

The Langmuir isotherm model assumes that the adsorption sites on the surface of the monolayer are uniform and equivalent, with no interaction between adsorbate molecules at adjacent locations, which is expressed as^40^:

9 $$\frac{{C}_{e}}{{q}_{e}}=\frac{{C}_{{\rm{e}}}}{{q}_{m}}+\frac{1}{{q}_{m}{K}_{L}}$$

where *q*_*m*_ (mg/g) is the maximum adsorption capacity, and *K*_*L*_ (L/mg) is the Langmuir constant associatd with the affinity of binding site and is also a measure of the free energy of adsorption.

The Freundlich isotherm model is used to depict the adsorption on an energy heterogeneous surface, and could be expressed as^41^:

10 $$ln{q}_{e}=ln{K}_{F}+\frac{1}{n}ln{C}_{e}$$

where *n* is the heterogeneity factor indicating the adsorption strength of the adsorbent, and *K*_*F*_ (mg/g∙(L/mg)^1/n^) is the constant in connection with the adsorption capacity.

Tempkin and Pyzhev assumed that some indirect adsorbate/adsorbate interactions had effect on adsorption isotherms and suggested that the adsorption heat of all the molecules in the layer would decrease linearly with coverage due to these interactions. The Tempkin isotherm has been used as below:

$${q}_{e}=\frac{RT}{b}lnA{C}_{e}$$

$${q}_{e}=\frac{RT}{b}lnA+\frac{RT}{b}ln{C}_{e}$$

$$B=\frac{RT}{b}$$

A plot of *q*_*e*_ versus ln*C*_*e*_ could determine the constants *A* and *B*. The constant B is related to the adsorption heat^42^.

The D-R empirical equation put forward by Dubinin and Radushkevich, has been widely employed to depict the gases and vapours adsorption on microporous solids. In the case of liquid phase adsorption, several researches have indicated that the adsorption energy can be estimated via D-R equation. Assuming only monolayer adsorption occurs in micropores adsorption and the D-R equation is applicable, the adsorption capacity per unit surface area of the adsorbent at equilibrium, *q*_*e*_, can be described as^43^:

$${q}_{e}={q}_{0}\exp (\,-\,B{\varepsilon }^{2})$$

$$\varepsilon =RT\,\mathrm{ln}(1+\frac{1}{{C}_{e}})$$

where *B* is the constant related to the adsorption energy, *q*_0_ is the ultimate capacity per unit area of adsorbent micropores, and *ε* is the Polenyi potential. The most probable adsorption energy, *E*, has been shown as:

$$E={(2B)}^{-\frac{1}{2}}$$

**Figure 11:**
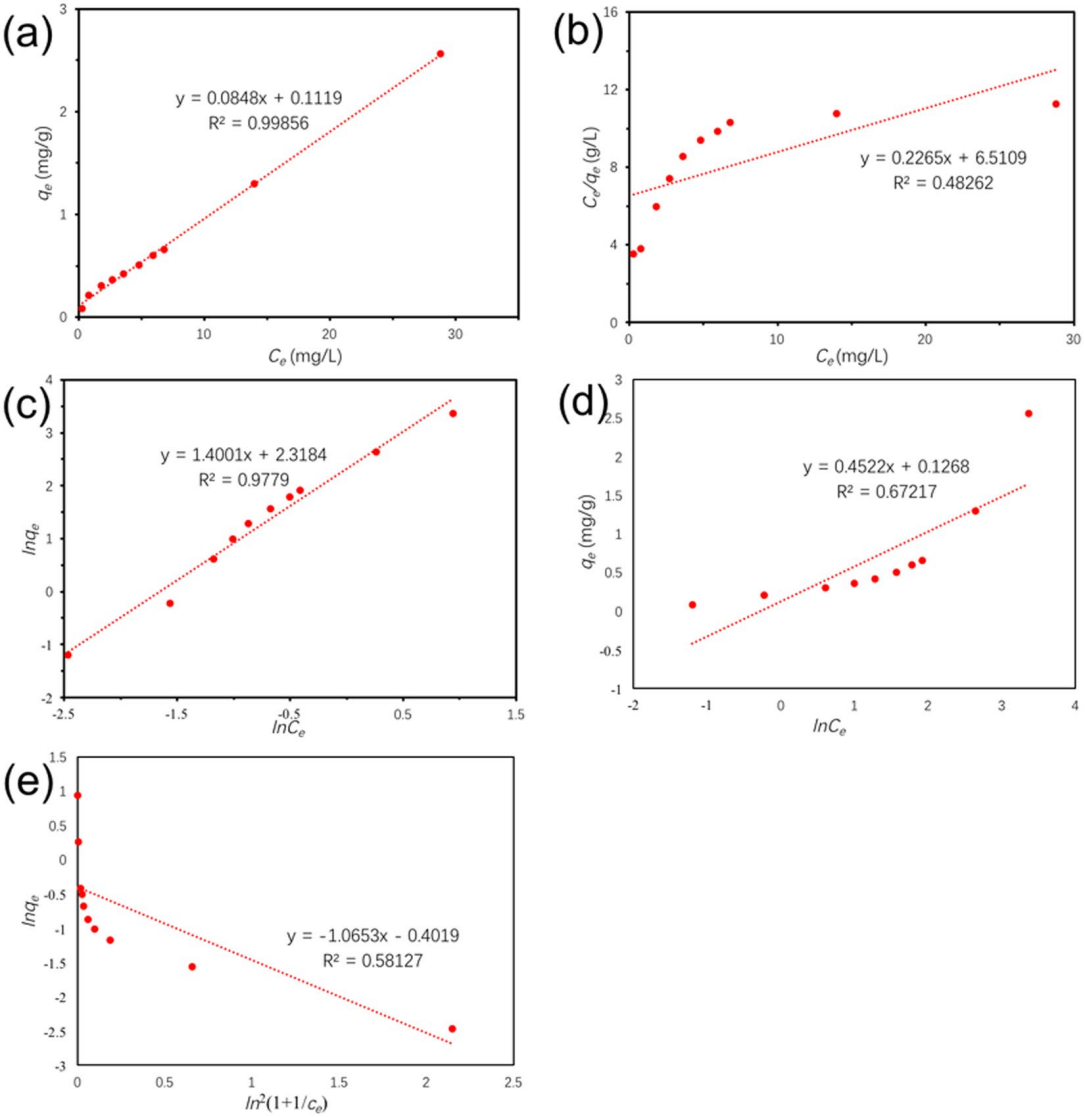
Linear (**a**), Langmuir (**b**), Freundlich (**c**), Tempkin (**d**) and D-R (**e**) plots of isotherms.

As Fig. 11 depicted, the linear, Langmuir, Freundlich, Tempkin and D-R isotherm models were used to fit TC adsorption data onto Ben/Rm/Ps-op. The parameter values were based on the regression of the isotherm equations and were summarized in Table 7. The results showed that Langmuir isotherm model was not suitable for the adsorption, demonstrating that the adsorption of TC by Ben/Rm/Ps-op was not monolayer adsorption, neither Tempkin nor D-R isotherm models. Whereas, linear isotherm model was suitable for the determination of data due to the higher correlation coefficient *R*^2^ (0.98689) than *R*^2^ (0.95035) of Freundlich. For linear model, the coverage of monolayer and the initial amount of multilayer adsorption appear to be superimposed. Since there is no platform in linear model, it indicates that the adsorption does not reach saturation, and the multilayer adsorption thickness seems to increase indefinitely.

**Table 7:** Isotherm model parameters for adsorption of TC by Ben/Rm/Ps-op ceramsite.

Model	Parameter	Ben/Rm/Ps-op ceramsite
linear model	*Kd* (L/g)	0.0848
	*R*^2^	0.99856
	*q*_*m*_ (mg/g)	2.55414
Langmuir model	*q*_*m*_ (mg/g)	4.41501
	*K*_*L*_ (L/mg)	0.034788
	*R*^2^	0.48262
Freundlich model	*K*_*F*_ (mg/g∙(L/mg)^1/n^)	10.1594
	*n*	0.71423
	*R*^2^	0.9779
Tempkin model	*A*	1.3237
	*B*	0.4522
	*R*^2^	0.6722
D-R model	*q*_0_(mg/g)	0.6690
	*B* (*J*^*−*2^)	1.8*10^−7^
	*E* (*J*)	1.67*10^3^
	*R*^2^	0.5813

#### Adsorption thermodynamics

**Figure 12:**
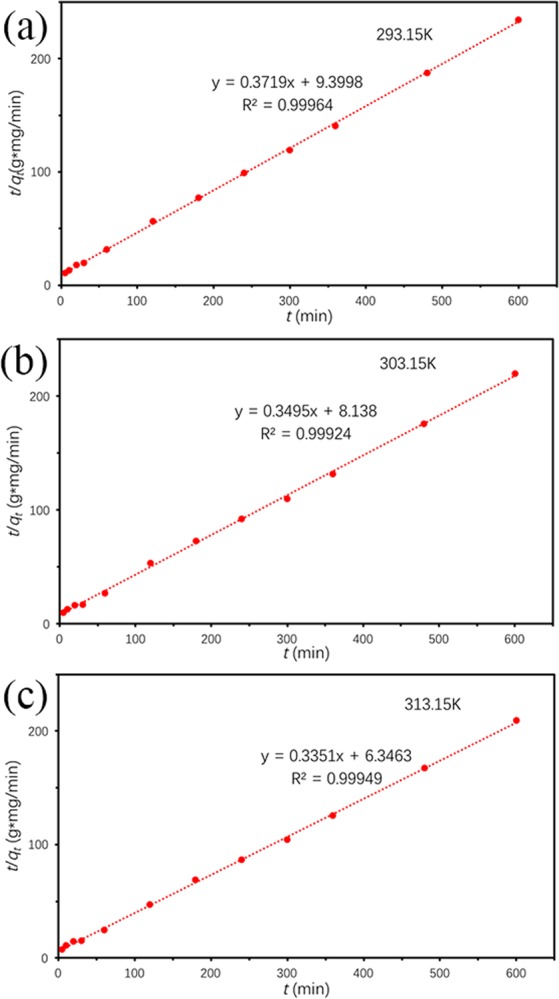
Pseudo-second-order model under 20 °C (293.15 K) (**a**), 30 °C (303.15 K) (**b**) and 40 °C (313.15 K) (**c**).

**Figure 13:**
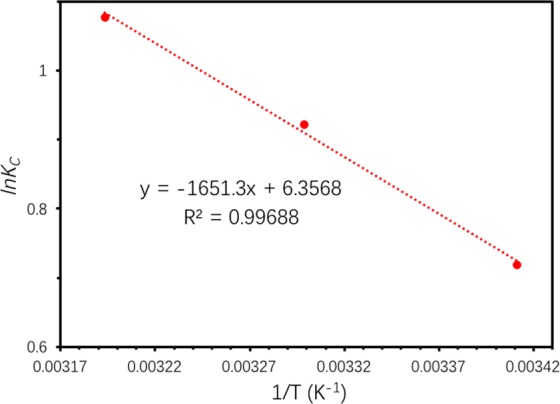
Plot of ln*Kc* versus 1/T for TC adsorption.

**Figure 14:**
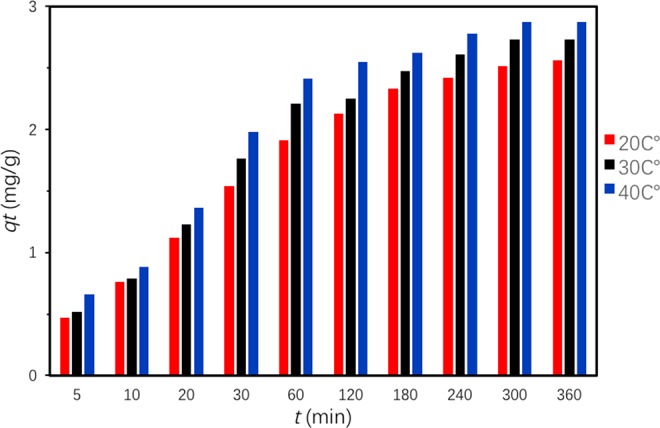
Adsorption Kinetics at 20 °C (**a**), 30 °C (**b**) and 40 °C (**c**).

Thermodynamic properties of TC onto Ben/Rm/Ps-op were further investigated, which could be described via Gibb’s free energy (*ΔG*°), enthalpy (*ΔH*°) and entropy (*ΔS*°). The thermodynamic were estimated using the following relations^38,44^:

11 $${K}_{C}=\frac{{C}_{Ae}}{{C}_{e}}$$

12 $$\varDelta {G}^{^\circ }=-\,{\rm{RT}}ln{K}_{c}$$

13 $$ln{K}_{{\rm{c}}}=-\,\frac{\varDelta {H}^{^\circ }}{RT}+\frac{\varDelta {S}^{^\circ }}{R}$$

where *R* (8.314 J/K mol) is the gas constant; *T* (K) is temperature; *K*_*c*_ is the equilibrium constant; *C*_*e*_ is the equilibrium concentration of TC in the solution (mg/L); and *C*_*Ae*_ is the amount of adsorbed TC on the adsorbent at equilibrium (mg/L). *C*_*Ae*_ and *C*_*e*_ are obtained from *q*_*e*_ values of the pseudo-second-order model (Fig. 12). Δ*S*° and Δ*H*° were acquired from the slope and intercept of linear plot of ln*K*_c_ versus 1/T according to Eq. (13) (Fig. 13). Figure 14 described adsorption capacity of ceramiste at different temperatures, and demonstrated that increasing temperature would promote adsorption capacity of TC by ceramsite. Table 8 presented the thermodynamic parameters at different temperatures. The negative values of Δ*G*° (−1.751, −2.322 and −2.806 kJ/mol for 293.15, 303.15 and 313.15 K respectively) suggested that the adsorption of TC onto Ben/Rm/Ps-op was spontaneous and thermodynamically favorable. Besides, Δ*G*° value decreased with an increase in temperature, indicating that the spontaneous nature of the adsorption was inversely proportional to the temperature and higher temperature enhanced the adsorption^45^. Moreover, the positive value of Δ*H*° suggested that the adsorption of TC onto Ben/Rm/Ps-op was an endothermic process. The positive value of Δ*S*^0^ revealed increased randomness at the solid/solution interface during the adsorption of TC onto Ben/Rm/Ps-op^46^.

**Table 8:** Thermodynamic parameters at different temperatures (293.15 K, 303.15 K and 313.15 K).

Temperature (K)	*K*_*c*_	*ΔG*^0^ (kJ/mol)	*ΔH*^0^ (kJ/mol)	*ΔS*^0^ (J/molK)	*R*^2^
293.15	2.0509	−1.751	13.728	52.8504	0.99688
303.15	2.5126	−2.322			
313.15	2.9377	−2.806			

#### Effects of pH, Ben/Rm/Ps-op dosage and ionic strength

**Figure 15:**
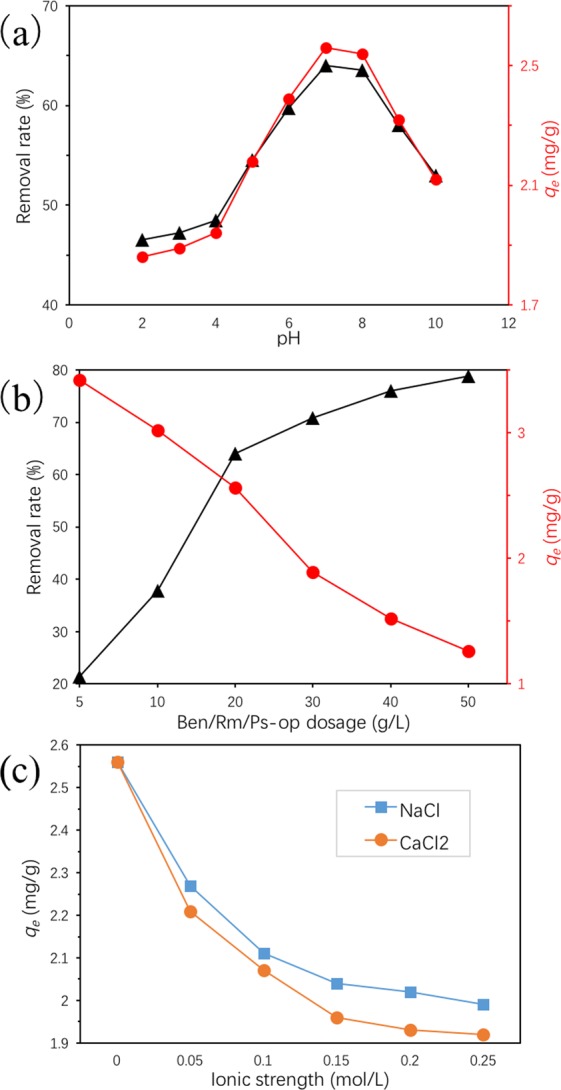
Effects of pH (2–10) (**a**), Ben/Rm/Ps-op dosage (5–50 g/L) (**b**) and ionic strength (0–0.25 mol/L) (**c**) on the adsorption capacity and removal rate of tetracycline (adsorption condition: *C*_0_ = 80 mg/L, *V* = 50 mL, speed = 160 rpm, *T* = 20 °C, pH = 7, *t* = 12 h).

For the effect of pH on adsorption, the adsorption capacity and removal rate of TC increase within pH 2–7, and decrease during pH 7–10 (Fig. 15a). Under strong acid condition (pH 2–4), TCH^3+^ is the main form of TC, which could be combined with silicon dioxide with negative charge in ceramsite. While hydrogen ions in the solution are more competitive than TC in binding to the ceramisite voids at the same time. So the amount of TC adsorbed by ceramisite (from 1.86 to 1.94 mg/g) and removal rate (from 46.5% to 48.5%) did not increase significantly in the range of pH 2–4. With increasing pH from 4 to 7, the adsorption capacity of TC sharply increased by 0.62 mg/g and the removal rate climbed to 64% at pH 7. This because H^+^ decreased in an order of magnitude, and the adsorption of TC by ceramsite increased obviously. However, from pH 7 to pH 10, the removal rate of TC by a large margin reduced to 53% with an adsorption capacity of 2.12 mg/g. During this period, the morphology of TC gradually changed from TCH_2_^°^ to TCH^−^, and the negative charge ratio on the surface of ceramsite increased, which were unfavorable to the adsorption of TC by ceramsite. This result indicates the adsorption capacity of Ben/Rm/Ps-op will be largely impacted by pH, and the adsorption performance was excellent at the neutral condition.

Besides, the adsorption of TC was conducted in the presence of different dosage of Ben/Rm/Ps-op. As Fig. 15b described, the removal rate of TC significantly increased from approximately 21.38% to 78.75% with increasing Ben/Rm/Ps-op dosage from 5 to 50 g/L. However, the TC adsorption capacity gradually decreased from approximately 3.42 to 1.26 mg/g. Given the efficiency and economy of such operation, the optimum Ben/Rm/Ps-op dosage is 20 g/L, under which both the adsorption efficiency and capacity were kept high.

In addition, adsorption experiments on the effect of ionic strength were conducted using 80 mg/L TC solution containing 0–0.25 mol/L NaCl or CaCl_2_ at pH = 7 and the temperature of 20 °C. Figure 15c describes the adsorption behavior of TC versus ironic strength. The existence of NaCl (or CaCl_2_) decreases the adsorption capacity of TC onto *Ben/Rm/Ps-op*, which may be due to the competitive effect between Na^+^ (or Ca^2+^) and TC on the adsorption sites. Parolo *et al*. observed that it can be explained that metal cations in solution could easily chelate with TC^47^, and electrolyte can produce electrostatic shielding effect, thus affect adsorption^48,49^. In addition, increasing Na^+^ (or Ca^2+^) concentration can bring in the contraction of adsorbent pores, leading to that some adsorbate could not enter into pores, and the reduction of surface adsorption sites of *Ben/Rm/Ps-op*^50–52^. Further, it is clear that NaCl, a univalent electrolyte, had less negative impact on TC adsorption than a divalent CaCl_2_ under identical conditions. Thus, it can be concluded that coexisting ions had adverse effect for TC adsorption onto *Ben/Rm/Ps-op*.

#### Dynamic adsorption of TC

**Figure 16:**
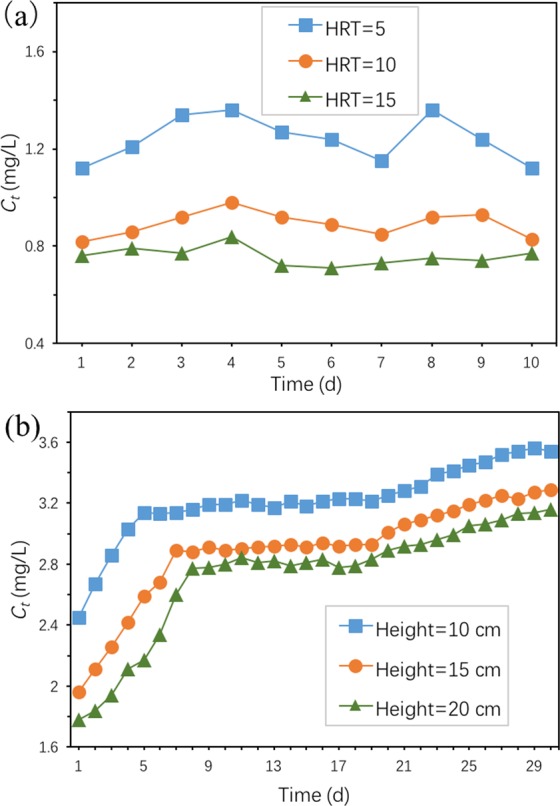
Dynamic adsorption of TC by Ben/Rm/Ps-op ceramsite packed column: (**a**) effect of HRT, (**b**) effect of packing height (adsorption condition: *C*_0_ = 4 mg/L, ceramic packing height = 30 cm, and HRT = 0.5 h for (**b**)).

The effect of hydraulic retention time (HRT) and packing height on TC removal were investigated, and the result was shown in Fig. 16. It can be seen from the figure that HRT had a great influence on the removal of TC by Ben/Rm/Ps-op. When HRT = 5, 10 and 15 h, the average removal rates of TC by ceramsite got to 69.0%, 77.7% and 81.1% respectively. With the increase of HRT, the removal rate of TC by ceramsite increases. The reason is that the increase of retention time of solution through the packed column will lead to more sufficient contact reaction between ceramsite and TC, which makes the total amount of TC adsorbed by ceramsite increase. In addition, with the increase of HRT, the amount of TC adsorbed by ceramsite increases, but the degree of increase decreases (77.7–69.0% > 81.1–77.7%). This could be interpreted as that with the prolongation of adsorption time, the adsorption sites decrease and the adsorption difficulty increase. Since the HRT of a CWs system is usually longer than 3 days^53^ and the removal efficiency have already reached 81.1% at HRT = 15 h, the Ben/Rm/Ps-op has the high potential to effectively remove TC as the CWs substrate under a dynamic flow condition.

Figure 16b presents TC concentration of Ben/Rm/Ps-op packed column at different packing heights. The TC concentration at different packing heights showed a similar change trend versus the operation time, i.e., rapidly increasing in the initial days, then reaching a relatively stable level, and gradually increasing in the late stage. However, the initial rapidly increasing takes different time. TC concentration quickly increase in the initial 5, 7 and 8 days, respectively for height = 10, 15, 20 cm. This may be attributed to that with the increase of the quality of adsorbent, the adsorption sites on the surface of adsorbent increase, which lead to prolonging the adsorption time of TC. We could also see in Fig. 16b, the lower section played the key role in the adsorption of TC, and it also arrive at saturation first during the continuously operation.

### Regeneration of Ben/Rm/Ps-op

**Figure 17:**
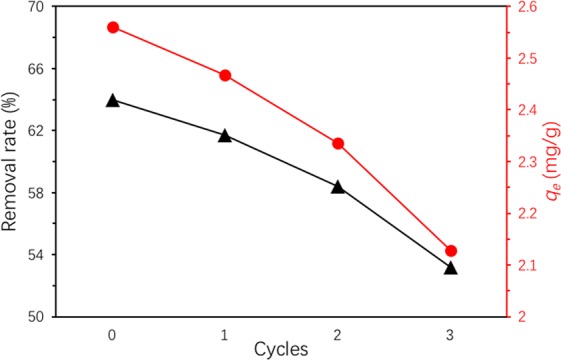
TC removal of recycled Ben/Rm/Ps-op ceramsite (adsorption condition: *C*_0_ = 80 mg/L, *V* = 50 mL, speed = 160 rpm, *T* = 20 °C, pH = 7, *t* = 12 h).

Seen in Fig. 17, good adsorption capability of the regenerated adsorbent was still retained after three rounds of sorption-desorption cycles. In addition, TC removal rates for repeated three times were 61.7%, 58.4% and 53.2% respectively. The decrease (2.3–5.2% in every cycle) of removal rate might have been owing to the loss of irreversible occupation of partial-adsorption sites^54^. Nevertheless, the adsorption amount of ceramsites still remained at a high value (2.13 mg/g, *C*_0_ = 80 mg/L) after three consecutive cycles, suggesting the high reusability capability and stability of Ben/Rm/Ps-op for TC removal.

## Conclusions

In this study, a kind of CWs Ben-Rm-Ps ceramsite was prepared to remove TC in effluent. Ben/Rm/Ps-op ceramsite was prepared with the condition Ben: Rm: Ps = 4:1:0.9, preheating temperature = 240 °C, preheating time = 20 min, calcining temperature = 1150 °C, and calcining time = 14 min, which possessed microporous structure and low heavy metal leaching toxicity. The second-order kinetics and linear isothermal model can well simulate the adsorption of TC by Ben/Rm/Ps-op, and the maximum adsorption capacity can reach for 2.5602 mg/g. In addition, TC adsorption onto Ben/Rm/Ps-op was demonstrated a spontaneous endothermic process and higher temperature enhanced the adsorption. Further, Ben/Rm/Ps-op has been also proved the high potential to effectively remove TC as the CWs substrate under a dynamic flow condition and high reusability capability and stability for TC removal under batch tests.

This study combines basic theory and engineering application research, and has important value for the research and development of CWs matrix filler. The research results of this subject will provide an important reference for the research and application of artificial calcined ceramsite as a light aggregate in water pollution control. In addition, the preparation and application of new ceramsite matrix can not only enhance the pollutant removal function of CWs, but also utilize solid wastes such as red mud and biomass. Therefore, the research and development of ceramsite products also has comprehensive economic, social and environmental benefits.
